# Editorial: A holistic approach to oral–systemic health care: integrating natural and modern therapies

**DOI:** 10.3389/fdmed.2026.1908324

**Published:** 2026-07-29

**Authors:** Mario Taba, Ammaar H. Abidi, Kavita Chandrasekaran

**Affiliations:** 1 University of São Paulo, São Paulo, Brazil; 2 Deparment of Biomedical Sciences and Department of Oral Health Sciences, Lincoln Memorial University College of Dental Medicine, Knoxville, TN, United States; 3 All India Institute of Medical Sciences Bibinagar, Hyderabad, India

**Keywords:** adjunctive therapy, antimicrobial, holistic approach, inflammatory mediators, oral healh, phytotherapy, plant extract

## Introduction

The integration of natural products and herbal medicine into dentistry represents a rapidly expanding frontier in oral health research, bridging traditional practices with evidence-based clinical science (1). This Research Topic of recent studies highlights the diverse therapeutic potential of biological extracts across multiple dental disciplines, including preventive dentistry, endodontics, periodontics, and oral medicine. Viewed collectively, these contributions move oral–systemic medicine beyond isolated product evaluation and toward a translational framework in which biologically active natural compounds are examined for mechanism, clinical relevance, safety, and integration with standard care. A recurring theme is that natural therapies may have their greatest value as adjuncts that support antimicrobial control, modulation of inflammation, tissue preservation, and symptom management rather than as direct replacements for established dental treatments.

*In vitro* and ultrastructural analyses evaluate how botanical extracts interact with the protective dental pellicle and disrupt the cell walls of virulent endodontic pathogens. Building upon these mechanistic foundations, preclinical systematic reviews synthesize laboratory and animal model data to evaluate the viability of natural alternatives for critical applications, such as preserving periodontal ligament cells during tooth avulsion and managing neuropathic orofacial pain.

The translational value of these therapies is established through human clinical evidence, including randomized controlled trials and network meta-analyses. These clinical studies directly compare herbal formulations—ranging from single-botanical essential oils to complex traditional solutions—against established comparator therapies for widespread conditions like gingivitis, chronic periodontitis, and oral lichen planus. These papers suggest that selected natural products may provide clinically meaningful adjunctive benefits, although claims of comparable efficacy to synthetic pharmaceuticals should be interpreted cautiously because much of the current evidence remains preclinical, short-term, or derived from small clinical studies.

By critically evaluating both the biological mechanisms and clinical limitations of these therapies, this Research Topic shows the evolving role of phytotherapy as a viable, accessible, and scientifically validated adjunct in modern comprehensive dental care; it emphasizes that these natural therapies are intended to complement—not replace—established standard care treatments.

## Strengths and limitations

A primary strength of these compiled studies is their dual validation of anti-inflammatory and antimicrobial properties across different clinical modalities. For instance, the down-regulation of pro-inflammatory cytokines (TNF-α, IL-1β, IL-6) and hs-CRP observed by Shen et al. when examined adjunctive use of a traditional Xipayi Gingival Solution with minocycline in chronic periodontitis patients aligns conceptually with the symptomatic relief and pain reduction reported in Wang's network meta-analysis of oral lichen planus (Shen et al., Wang et al.).

Similarly, the structural membrane disruptions caused by Phyllanthus niruri against common endodontic pathogens (E. faecalis, P. gingivalis, T. denticola) *in vitro* presented by Shivakumar et al. offer a mechanistic parallel to the significant reduction of microbial loads achieved clinically by tea tree oil in Menat et al. exploratory randomized, triple-blind clinical trial comparing 1% TTO mouthwash with 0.12% CHX in adults with plaque-induced gingivitis. In addition, the effects of wild strawberry leaf (WSLE) extract on initial pellicle ultrastructure and enamel calcium release was investigated by Frey et al. Contrary to expectations for polyphenol-rich rinses, WSLE did not thicken or densify the dental pellicle (Shivakumar et al., Menat et al., Frey et al.).

The clinical translation of natural interventions within dental medicine shows varying levels of evidence; while Chhabra et al. utilized a quality assessment tool (QUIN) to confirm that 10% propolis effectively preserves avulsed teeth over standard alternatives like milk or coconut water, Jasim et al. reported that despite positive trends in pain reduction, the current preclinical evidence for herbal remedies in trigeminal neuropathy remains of low certainty due to high risks of bias and model variability (Chhabra et al., Jasim et al.).

This Research Topic advances the field of oral-systemic medicine by shifting the narrative of natural products from “alternative remedies” to a scientific path for multi-target therapeutic adjuncts. By targeting microbial and inflammatory challenges, these therapies support the biological rationale that oral tissue health is linked to systemic homeostasis (2).

Despite these advances, the widespread clinical translation is still evolving. A common gap exists in product standardization; natural compounds vary heavily by geographical origin, harvest season, and extraction technique. Until researchers establish strict chemical profiling and uniform active-ingredient dosing, replicating these positive outcomes across diverse populations will remain a challenge.

## Recommendations for future research

Across the Research Topic, several shared strengths emerge: biologic plausibility, patient-centered interest in better-tolerated therapies, and early evidence of antimicrobial, anti-inflammatory, mineral-modulating, or analgesic activity. However, the same body of work also highlights persistent limitations, including heterogeneity in formulations, dosing, outcome measures, follow-up duration, and study quality. These gaps limit direct clinical translation and underscore the need for standardized preparations, larger controlled trials, longer follow-up, and clearer safety monitoring.

These seven contributions (Table 1) converge on several practical and conceptual themes. Natural products and traditional extracts frequently demonstrate biological activity—antimicrobial, anti-inflammatory, anti-nociceptive, or mineral-modulating—that can complement modern therapeutics across diverse oral conditions from traumatic injuries to chronic inflammatory diseases (2). Therefore, standardization for clinical translation requires uniform concentrations, preparation methods, vehicles, dosing schedules, and validated outcome measures. Also, short-term tolerability is favorable, but long-term effects and interactions with standard therapies require systematic evaluation.

**Table 1:** Overview of contributing papers.

Research category	Study topic & authors	Methodology & key measures	Main findings & conclusions
*In Vitro* & Ultrastructural Studies *(Basic Science)*	Fragaria vesca Leaf Extract (WSLE) & Dental Pellicle (Frey et al.)	TEM and AAS analysis of initial pellicle ultrastructure and enamel calcium release.	WSLE did not thicken the pellicle; increased calcium release under acidic challenge; may act as an enamel calcium source.
	Phyllanthus niruri Antimicrobial Mechanism (Shivakumar et al.)	SEM and TEM characterization against *E. faecalis*, *P. gingivalis*, and *T. denticola*.	Demonstrated bacteriostatic activity and cell membrane deformation; potential adjunct intracanal medicament at 250 mg/mL.
Preclinical & *in vitro* Reviews *(Evidence Synthesis)*	Propolis Storage Medium for Avulsed Teeth (Chhabra et al.)	PRISMA systematic review evaluated by QUIN tool for PDL cell viability.	10% propolis maintained higher PDL cell viability than HBSS, milk, coconut water, or pomegranate juice.
	Herbal Meds for Trigeminal Neuropathy (Jasim et al.)	Systematic review of rodent models for post-traumatic trigeminal neuropathy (PTTN).	Herbs increased pain thresholds, but high study bias and heterogeneity mean overall efficacy remains inconclusive.
Clinical Trials & Meta-Analyses *(Human Data)*	Tea Tree Oil (TTO) vs. Chlorhexidine (CHX) (Menat et al.)	15-day exploratory randomized, triple-blind clinical trial for plaque-induced gingivitis.	Both reduced plaque and bacteria; CHX had higher bacterial reduction, but TTO showed fewer adverse effects.
	Xipayi Gingival Solution for Periodontitis (Shen et al.)	Randomized clinical trial of combined Xipayi and minocycline therapy.	Combined therapy reduced periodontal indices, cytokines, and oxidative stress better than minocycline alone.
	TCM Biological Extracts for Oral Lichen Planus (Wang et al.)	Network meta-analysis of 22 randomized controlled trials using SUCRA rankings.	TCM extracts reduced pain; chamomile, echinacea, honey, and curcumin ranked highest for overall symptom relief.

Key findings and conclusions of contributing papers.

Clinicians should interpret the current evidence as promising but not definitive. Natural and traditional therapies may be considered as adjunctive options when supported by patient preference, product quality, safety data, and appropriate clinical judgment; however, they should not replace established preventive, periodontal, endodontic, or pharmacologic standards of care. Shared decision-making should include discussion of the strength of evidence, possible adverse effects, product variability, and the limited long-term data available for many interventions.

## Conclusions

The articles in this Research Topic highlight the promise of integrating natural and traditional therapies into modern dental care, particularly as adjuncts for antimicrobial control, modulation of inflammation, tissue preservation, and symptom management. At the same time, the Research Topic emphasizes that evidence-based integration requires standardized formulations, rigorous clinical trials, mechanistic clarity, and long-term safety data. Therefore, integrating these therapies into evidence-based clinical practice is recommended only after high-quality clinical validation. For oral–systemic medicine, the central contribution of this work is not simply that natural therapies may be beneficial, but that they provide a growing translational model for evaluating how biologically active compounds can complement patient-centered, evidence-based dental care.
